# Cold Hardiness and Supercooling Capacity in the Overwintering Larvae of the Codling Moth, *Cydia pomonella*

**DOI:** 10.1673/031.010.8301

**Published:** 2010-06-30

**Authors:** Abbas Khani, Saeid Moharramipour

**Affiliations:** Department of Entomology, Faculty of Agriculture, Tarbiat Modares University, P.O. Box 14115-336, Tehran, Iran; ^1^ Current address: Department of Plant Protection, Faculty of Agriculture, University of Zabol, Zabol, Iran

**Keywords:** codling moth, diapause, freeze-intolerant insect, low temperature

## Abstract

The codling moth, *Cydia pomonella* L. (Lepidoptera: Tortricidae), a worldwide apple pest, is classified as a freeze-intolerant organism and one of the most cold-tolerant pests. The objectives of this study were to examine the supercooling point of overwintering and non-diapausing larvae of *C. pomonella* as an index of its cold hardiness, and to assess larval mortality following 24 h exposure to extreme low temperatures ranging from -5 to -25°C. The mean (±SE) supercooling point for feeding larvae (third through fifth instars) was -12.4 ± 1.1°C. The mean supercooling point for cocooned, non-diapausing larvae (i.e., non-feeding stages) decreased as the days that the arvae were cocooned increased and changed between -15.1 ± 1.2°C for one to two day cocooned arvae and -19.2 ± 1.8°C for less than five day cocooned larvae. The mean (±SE) supercooling point for other non-feeding stages containing pupae and overwintering larvae were -19.9 ± 1.0°C and -20.2 ± 0.2°C, respectively. Mean supercooling points of *C. pomonella* larvae were significantly lower during the winter months than the summer months, and sex had no effect on the supercooling point of *C. pomonella* larvae. The mortality of larvae increased significantly after individuals were exposed to temperatures below the mean supercooling point of the population. The supercooling point was a good predictor of cold hardiness.

## Introduction

Cold hardiness and diapause are essential components of winter survival for most insects (Denlinger 1991). Insects from temperate, polar, and high-altitude environments must survive the low temperatures they encounter in their habitat, and a large literature has been developed regarding this important component of insect life histories (Somme 1999). Cold-tolerance strategies of insects have generally been divided into two major categories: freeze-tolerant insects tolerate the formation of extra-cellular ice within the body, whereas freeze-intolerant insects avoid the lethal effects of freezing by lowering the temperature at which the spontaneous freezing of body water occurs (Baust and Rojas 1985; Zachariassen 1985). This value is termed “the supercooling point” and is experimentally determined by detecting the released latent heat of fusion as body water freezes (Somme 1999).

The supercooling point represents the lowest lethal temperature for freeze-intolerant individuals, although death may also occur at temperatures above this point as a result of chill injury (Lee 1991; Lee and Denlinger 1985). Therefore in such instances, the supercooling point is not a reliable indicator of cold hardiness and further experiments must be carried out to determine its relationship to mortality at low temperatures (Bale et al. 1988). In addition to the supercooling point, lowest lethal temperature and lethal time have been used as indices of cold hardiness (Watanabe 2002).

The codling moth, *Cydia pomonella* L. (Lepidoptera: Tortricidae), is the most serious worldwide apple pest (Barnes 1991; Dorn et al. 1999). *C. pomonella* cause significant economic losses in apple orchards of Iran (Rajabi et al. 1978; Akrami 1984; Saeb 1994), and it attacks other fruits including pears, quinces, apricots, plums, and walnuts. The species has been intensively studied over the last hundred years in Europe, Asia, America, and other parts of the world where it is present. *C. pomonella* passes the winter as a full-grown larva in diapause inside a thick, silken cocoon and is able to withstand cold temperatures (Newcomer 1920; Yothers and Carlson 1941; Siegler 1946; Geier 1963; MacPhee 1964). Studies on cold hardiness of *C. pomonella* were established from the early part of this century (Newcomer 1920; Cory and Sanders 1932; Siegler 1946; Kot 1958), and controlling *C. pomonella* larvae with low-temperature storage as a quarantine treatment was studied in apples and stone fruits (Newcomer 1936; Moffitt and Albano 1972; Yokoyama and Miller 1989; Toba and Moffitt 1991). The studies of some researchers explained that *C. pomonella* was freeze-intolerant with minimum whole body supercooling points between -25 and -30°C (Siegler 1946; Minder et al. 1984; Neven 1999). Minder et al. (1984) observed that the highest resistance to cold was when the average supercooling point was minimized. Concentrations of a known cryoprotectant, glycerol, increased concurrently with seasonal decreases in the supercooling point of *C. pomonella* larvae (Minder et al. 1984). However, our recent study showed that trehalose was found to be the major cryoprotectant in overwintering larvae of *C. pomonella* (Khani et al. 2007). Neven (1999) reported that *C. pomonella* might be a freezeintolerant insect with a degree of chill tolerance, but did not find a significant difference in the whole body levels of trehalose or glycerol between diapause-induced and non-diapausing larvae of *C. pomonella.* However, cold hardiness may be affected by several other factors including geographic location, environmental conditions, developmental stage, sex, and age (Somme 1982; Turnock et al. 1990; Renault et al. 2002).

The objective of the current study was to measure the effect of feeding, season, and sex on the supercooling point. The acquired cold hardening was investigated by the quantitative assessment of supercooling points and survival at subzero temperatures.

## Materials and Methods

### Determination of supercooling points

The relative cold hardiness of *C. pomonella* larvae was examined by measuring the supercooling point using surface contact thermometry. Individuals were attached to a thermocouple (NiCr-Ni probe) using plastic glue tape. Insect-thermocouple arrangements were placed inside a programmable refrigerated test chamber (Binder GmbH Bergstr., model MK 57, Germany), whose temperature was lowered at a rate of 0.5°C/min to -30°C. Temperatures were recorded with a four-channel data logger (Testo, model 177-T4) that transferred data at 30s intervals into a computer and data was read using Comsoft 3 software. The supercooling point of each insect was recorded as the lowest reading reached before the release of latent energy, shown as a sudden increase in the temperature during an otherwise decreasing trend (Lee 1991). Generally, *C. pomonella* larvae in nature are able to move immediately after exiting from their cocoon even at low temperatures. Likewise, at the time of supercooling point determination, spending a few minutes for attaching the thermocouple, the specimens were able to move and show vital signs.

### Effect of feeding status on supercooling points

Individuals used in this study were field-collected larvae from Tehran province (35° 55′ N, 50° 54′ E). Infested fruits were collected from the commercial apple orchards in early August 2006 and kept at cyclic regimes of temperature and a photoperiod similar to environmental conditions (cyclic regimes consisted of higher thermophase (30°C) and lower cryophase (20°C) temperatures), under a photoperiod of 16:8 L:D in a programmable refrigerated test chamber (Binder GmbH Bergstr., model MK 53, Germany). In addition, cardboard strips (which served as cocoon sites for fifth instars) were placed on top of the infested apples. The strips were removed every day, labeled, and held until desired days of development prior to treatment. Field-collected larvae were recognized to be in good health and without any infections to pathogens or parasites.

Actively feeding larvae (third through fifth instars) were obtained from infested apples in early August. Cocooned, non-diapausing larvae (fifth instars) and pupae were taken from the cardboard strips. Supercooling point measurements were recorded from 15 feeding larvae (pooled third through fifth instars), 15 non-diapausing larvae one to two days after cocooning, 10 non-diapausing larvae three to five days after cocooning, 10 non-diapausing larvae less than five days after cocooning, and 25 pupae. Data were not analyzed statistically because each stage of development was measured at separate times because of the difficulty of synchronizing the development of all stages tested.

### Effect of season and sex on supercooling points

The effect of season on the supercooling point of *C. pomonella* larvae was examined for populations from Tehran province, Iran. Larvae used in this study were collected from commercial orchards, 24 h before measuring the supercooling point and kept at environmental conditions. Supercooling points of non-diapausing larvae (fifth instars) taken from infested apple fruits were determined during August 2005. Natural diapausing (fifth instar) larvae were collected from cardboard bands which were placed around the trunks of apple trees in early September. Supercooling points of overwintering larvae were measured from September 2005 to March 2006. One day after collection, individuals were sexed and their supercooling points were determined. Male larvae could be distinguished from female larvae by the dark pigmented testicles shining through the integument of their back (Sieber and Benz 1978). For each date, supercooling-point measurements were determined from 20 to 40 individuals. Supercooling points were tested for normality using the Kolmogorov-Smirnov Test, a non-parametric test for the accuracy of data. The seasonal change of mean supercooling points of individuals was analyzed using one-way analysis of variance (ANOVA) with Tukey's studentized range test (honestly significant difference [HSD]) using SPSS version 13.00 for Windows. Month was the sole predictor used in the ANOVA models. The differences between the supercooling points of non-diapausing and overwintering larvae were analyzed using independent *t*-student tests. Data for sex comparisons within various dates were analyzed using the General Linear Model (GLM) Univariate procedure. For the sex comparison sex, collection date, and the interaction between sex and collection date were included in the model.

### Effect of subzero temperatures on larval mortality

The effects of subzero temperatures on the survival of *C. pomonella* larvae were examined for both male and female fieldcollected larvae. The fifth instars larvae used in this study were collected in August 2005 (non-diapausing) and also from September to March 2006 (overwintering larvae) from Tehran province, Iran. Groups of 10 larvae (20 larvae of each sex, at each temperature, for each date) were placed into 15 × 150 mm glass test tubes. Test tubes containing larvae were placed into a programmable test chamber and cooled at a rate of ≈0.5°C/min. Larvae were cooled to -5, -10, -15, -20, or 25°C. The larvae were held at these temperatures for 24 h then warmed to 25°C at a rate of 0.5°C/min and held at that temperature for 24 h. Thereafter, the numbers of live and dead larvae were counted. The larvae showing no movement were judged to be dead. A non-parametric test (Mann—Whitney U Test and Kruskal—Wallis Test) were used to test for differences in the proportionate mortality of each treatment.

The mortality data at given temperatures over the several months of testing were compared with the supercooling points of field-collected larvae (102 male and 128 female). The cumulative proportion of individual supercooling points was calculated by summing the number of larvae that supercooled at or above each 1-degree temperature step, and dividing each resulting sum by the total number of individuals measured.

## Results

### Effect of feeding status on supercooling points

**Figure 1.**
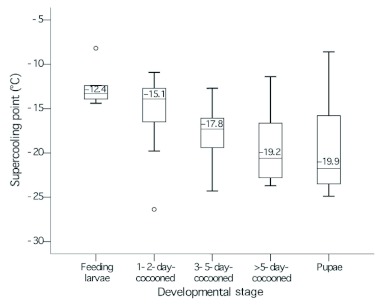
Effect of developmental stage (feeding status) on the supercooling points of *Cydia pomonella* larvae. The center bars of the box plots represent the median; the upper and lower ends of the boxes represent the 25th and 75th percentiles; the whiskers represent the minimum and maximum observed values that are not statistically outlying; circles represent outliers; and the data into boxes represent the mean. High quality figures are available online.

When the summer form larvae left the food source and spun a cocoon, whole body supercooling points decreased. In rank order, the mean (±SE) for feeding larvae (third through fifth instars), was -12.4 ± 1.1°C; one to two day cocooned, non-diapausing larvae, 15.1 ± 1.2°C; three to five day cocooned larvae, -17.8 ± 1.1°C; more than five day cocooned larvae, -19.2 ± 1.8°C; and pupae, 19.9 ± 1.0°C (Figure 1).

### Effect of season and sex on supercooling points

Diapause initiation had a significant effect on supercooling points. Non-diapausing larvae (fifth instars) had supercooling points from 8.2 to -26.4°C (mean = -16.3 ± 0.75), apart from feeding status, that were significantly higher than those of overwintering larvae (mean = -20.2 ± 0.25) (*t* = 5.87; *df* = 231; *P* < 0.0001). The mean supercooling point of overwinteing larvae significantly changed over time, from September 2005 through March 2006 (*F* = 6.24; *df* = 6, 194; *P* < 0.0001) (Table 1). The mean supercooling point decreased ≈6°C from August (non-diapause larvae; mean = -16.3 ± 0.75) to November 2005 (diapause larvae; mean = 22.5 ± 0.56).

The mean supercooling point of *C. pomonella* larvae was not significantly affected by sex (*F* = 0.00; *df* = 1, 224; *P* = 0.988) (Table 1). In addition, the interaction of sex and date was not significant (*F* = 0.166; *df* = 7, 208; *P* = 0.982), however, the effect of date was significant (*F* = 6.78; *df* = 7, 224; *P* < 0.0001) (Table 1).

### Effect of subzero temperatures on larval mortality

The mean percentages of mortality at 15°C/24h and -20°C/24h were significantly decreased over time, from August 2005 through March 2006 (Kruskal-Wallis Test, *P* < 0.01), the highest cold hardiness was observed from November through January (Tabel 1). At -20°C/24h, from October to February the males had a lower mortality than the females (*F* = 4.42; *df* = 1, 47; *P* = 0.043) (Table 1).

**Figure 2.**
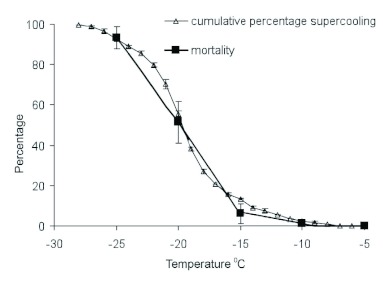
Mean (±SE) percentage of mortality and cumulative percentage of *Cydia pomonella* larvae supercooling at different subzero temperatures for field-collected individuals from August 2005 through March 2006. The mortality and supercooling point data is all the samples at a given temperature over the several months of testing. High quality figures are available online.

More than 98% of field-collected larvae survived at -10°C/24h; while more than 98% individuals died at -25°C/24h. Significant differences in the proportion of larval mortality among subzero temperatures were observed for field-collected individuals regardless of sex and date collection (Mann— Whitney U Test, *P* < 0.0001). The percentage of mortality increased significantly from lower than 10% to about 50% with decreasing exposure temperature from -15°C to -20°C) (Mann—Whitney U Test, *P* < 0.0001) (Figure 2.

**Table 1.**
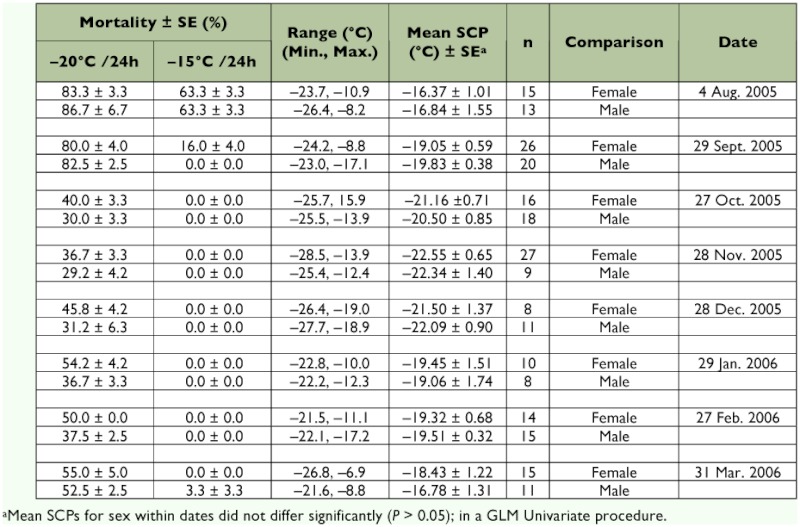
Effect of sex on the supercooling point (SCP) of *Cydia pomonella* larvae

The percentage of mortality in both sexes remained below 10% at temperatures higher than the mean supercooling point (Figure 2). After the mean supercooling point was surpassed, a significant increase in mortality (>50%) was observed for individuals from both sexes (Mann—Whitney U Test, *P* < 0.0001) (Figure 2). The curves for the cumulative percentage of individual supercooling points were consistently shifted in concert with the mortality curves (Figure 2).

## Discussion

The ability of *C. pomonella* to overwinter has received worldwide attention, (Newcomer 1920; Cory and Sanders 1932; Siegler 1946; Geier 1963; MacPhee 1964; Tomkins et al. 1987; Berankova et al. 1988). Reports of low overwintering mortality (Newcomer 1920; et Yothers and Carlson 1941; Kot 1958; Geier 1963; MacPhee 1964; Sato 1964; Berankova al. 1988) suggest that cold winter temperatures are not an important factor in regulating populations of *C. pomonella* from one year to the next. In this study, few mortalities were generally observed (<4%) in overwintering larvae collected in samplings during the winter. This high level of cold hardiness in overwintering larvae of *C. pomonella* might be dependent upon the ability of this insect to increase its cold hardiness (e.g., depress the supercooling point) during winter months, accompanied by the progress of diapause (Minder et al. 1984; Neven 1999; Khani et al. 2007). Our results indicate that the supercooling point of *C. pomonella*, as with other freeze-intolerant insects, significantly changes with the season. The mean supercooling point for field collected larvae in this study is similar to values previously reported for *C. pomonella* from the laboratory (Neven 1999) in the overwintering site in the field (Khani et al. 2007). In addition, it appears that developmental stage and feeding status may also have an effect on the supercooling point of this insect. The estimated mean supercooling point for feeding larvae was considerably higher than values determined for non-feeding stages containing cocooned larvae, overwintering larvae, and pupae of this species. The mean supercooling points for non-feeding stages (i.e., older cocooned larvae, overwintering larvae, and pupae) of *C. pomonella* remained below -18°C, while the mean supercooling points of the feeding stages (i.e., immature larvae) were warmer than -15°C. This pattern of higher supercooling points in feeding stages compared to the non-feeding stages has been identified previously (Koch et al. 2004; Carrillo et al. 2005; Khani et al. 2007). The low supercooling points of non-feeding stages are generally attributed to the fact that these stages have fewer ice nucleating agents 1999).

(INAs) compared to the feeding stages (Salt 1953; Leather et al. 1993). Also, the lower supercooling points for field-collected *C. pomonella* larvae during the winter may be partially attributed to an absence of food in the digestive tract (Lee et al. 1996; Neven Freeze-intolerant insects die when exposed to temperatures at or below the supercooling point, but some die at temperatures above the supercooling point because of chill injury (Lee 1991). However, our previous studies showed that *C. pomonella* was a chill tolerant insect with less than 5% mortality at 5°C/30d (Khani et al. 2007). In concert with the results of this study, it has been reported that *C. pomonella* larvae survived in the stored apple fruits for up to three months at 0–4 °C during post harvest conditions (Moffitt and Albano, 1972; Toba and Moffitt, 1991). For *C. pomonella*, the supercooling point appears to be a good indicator of cold hardiness when mortality is assessed after an exposure period of 24h. A high coincidence was observed between the decrease of the supercooling point and the increase of the survival rate in the overwintering larvae. Similar results have been observed for other freeze-intolerant insects; *Plodia interpunctella* (Hübner) (Carrillo et al. 2005) and *Harmonia axyridis* (Pallas) (Koch et al. 2004).

According to Bale (1991), freeze-tolerance or intolerance for an organism can be determined by exposing insects to temperatures above and below the mean supercooling point. By comparing the cumulative proportion at which individuals freeze to the rate of mortality at different subzero temperatures one can determine if a species is tolerant or intolerant of freezing (Koch et al. 2004; Carrillo et al. 2005). For *C. pomonella*, a similar gap between these two curves indicated that mortality occurred at or near larval supercooling point temperatures and that these larvae were freeze-intolerant. Also, the overwintering larvae could not tolerate temperatures lower than the mean supercooling point (e.g -25 °C used in this study) and death after the measuring of the supercooling point indicates that *C. pomonella* is a freeze-intolerant insect. Findings from this study showed similar trends in association of supercooling point and cold hardiness in overwintering *C. pomonella* larvae studied in 2004–2005 (Khani et al. 2007). Similar results have been observed for other freeze-intolerant insects (Coulson and Bale 1996; Needham et al. 1996; Jones and Kunz 1997).

The physiological mechanisms underlying the ability of *C. pomonella* larvae to depress supercooling points to temperatures of -25°C is known to some extent. The lowest mean supercooling point observed in the cocooned, non-diapausing larvae (-26.4°C) and in overwintering *C. pomonella* larvae (-28.5°C) may be partially due to an absence of food in the digestive tract. Neven (1999) suggested that diapause-induced *C. pomonella* larvae may empty their gut to reduce ice nucleators in preparation for diapause initiation. However, a possible production of antifreeze compounds such as alcohols and sugars also may be involved in the depression of the supercooling point. Neven (1999) also looked at differences in sex with trehalose, but found no difference. The lowest mean supercooling point of field-collected larvae also may corroborate previous observations where *C. pomonella* were able to depress their supercooling points by accumulation of glycerol as cryoprotectant (Minder et al. 1984). However, our recent study showed that overwintering larvae accumulated trehalose during winter and trehalose content synchronized seasonally with supercooling capacity in overwintering larvae of *C. pomonella* (Khani et al. 2007).

In this study, *C. pomonella* populations were studied in their natural habitats. Investigations in the microclimatic conditions experienced by larvae show the importance of the habitat and its role in ensuring survival at low temperatures. Climate change will lead to an improvement in winter survival, however, increased climatic variability might leave insects ill equipped to deal with the altered conditions and consequently increase mortality (Sinclair et al. 2003). Therefore, prediction of direct effects of climate on insects is important. It is clear that freeze avoidance is the most common strategy among insects (Sinclair et al. 2003). Environmental predictability has a larger influence on insect cold hardiness. In an unpredictable environment, freeze avoidance species display low supercooling points irrespective of season. As proposed by Sinclair et al. (2003), species that show pre-freeze mortality have a well—developed rapid cold hardening response which enables virtually instantaneous response to unpredictable events. Therefore, the significance of the freeze avoidance strategy and pre-freeze mortality in *C. pomonella* larvae, which experience unpredictable environments particularly in autumn and spring, could be elucidated.
